# *Trametes versicolor* (Synn. *Coriolus versicolor*) Polysaccharides in Cancer Therapy: Targets and Efficacy

**DOI:** 10.3390/biomedicines8050135

**Published:** 2020-05-25

**Authors:** Solomon Habtemariam

**Affiliations:** Pharmacognosy Research Laboratories & Herbal Analysis Services UK, University of Greenwich, Chatham-Maritime, Kent ME4 4TB, UK; s.habtemariam@herbalanalysis.co.uk; Tel.: +44-208-331-8302

**Keywords:** *Coriolus versicolor*, *Trametes*, cancer, polysaccharides, PSP, PSK, immunostimulation, adjuvant therapy

## Abstract

*Coriolus versicolor* (L.) Quél. is a higher fungi or mushroom which is now known by its accepted scientific name as *Trametes versicolor* (L.) Lloyd (family Polyporaceae). The polysaccharides, primarily two commercial products from China and Japan as PSP and PSK, respectively, have been claimed to serve as adjuvant therapy for cancer. In this paper, research advances in this field, including direct cytotoxicity in cancer cells and immunostimulatory effects, are scrutinised at three levels: in vitro, in vivo and clinical outcomes. The level of activity in the various cancers, key targets (both in cancer and immune cells) and pharmacological efficacies are discussed.

## 1. Introduction

**Figure 1:**
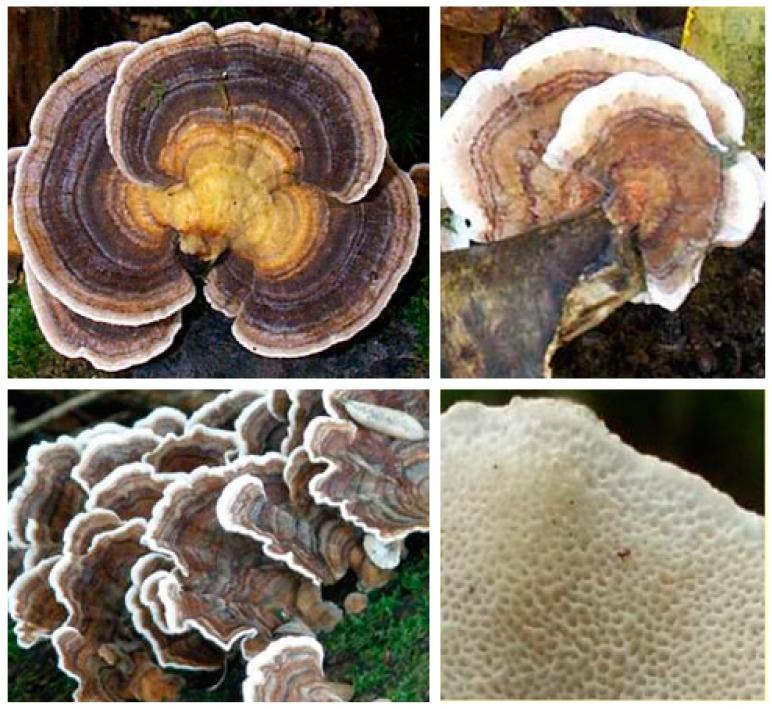
Morphological features of *Coriolus versicolor*. The various morphological features of the fungus grown in the UK are shown. While the upper surface shows concentric zones of colours (red, yellow, green, blue, brown, black, and white), the picture in the lower-right shows the polyporous nature of the underside portion of the fungus. Pictures are a kind gift of *first-nature.com* (https://www.first-nature.com/fungi/trametes-versicolor.php#distribution).

According to the recent WHO figure [1], cancer is the second most leading cause of mortality in the world and accounts for an estimated 9.6 million deaths in 2018. Most of the cancer death (~70%) occur in developing or the so-called low- and middle-income countries where access to modern medicines are not widely available. The common cancer deaths are from the lung, colorectal, stomach, liver and breast cancers, respectively, while other common cancers include prostate and skin cancers [1]. The major control measure for cancer is chemotherapy by using a variety of small molecular weight compounds and biological agents. As always, nature has its fair share of abundance as a source of these agents and drugs like paclitaxel (Taxol^®^), podophyllotoxin derivatives and vinca alkaloids (vinblastine and vincristine) are our excellent examples for potential exploration of more novel anti-cancer agents from higher plants. On the other hand, doxorubicin, daunomycin, mitomycin C, and bleomycin are good representative examples of anti-cancer agents explored from fungal sources, particularly *Streptomyces.*

In addition to their nutritional value, medicinal mushrooms have emerged in recent years not only as a source of drugs but also as adjuvants to conventional chemo- or radiation-therapy to either enhance their potency or reduce their side effects ( see [2] and references therein). In this regard, one of by far the best investigated medicinal mushroom in recent years is *Coriolus versicolor* (L.) Quél. *(Syn. Polyporus versicolor)* which is now known by its accepted scientific name as *Trametes versicolor* (L.) Lloyd (family Polyporaceae). Its most common name in the Western world is Turkey Tail, and its distinct morphological features include the concentric multicoloured zones on the upper side of the cap (no stalk) and spore-bearing polypores underside (Figure 1). The fungus is common in temperate Asia, North America and Europe, including the UK, where it has been recorded in all regions [3]. Its medicinal value as part of the Chinese traditional medicine dates back for at least 2000 years and includes general health-promoting effects [4], including endurance and longevity. Both in China and Japan, preparations such as dried powdered tea of the fungus are employed in traditional medicine practices. In this communication, the main components of the fungi, polysaccharides, that have given the fungi its medicinal value in cancer therapy are assessed by reviewing the chemistry, pharmacology and therapeutic potential at three levels: in vitro, in vivo and clinical studies. Readers should note that nearly all the published literature in this field is available under the name *Coriolus versicolor (Trametes versicolor*).

## 2. Overview of Chemistry

### 2.1. Small Molecular Weight Compounds

**Figure 2:**
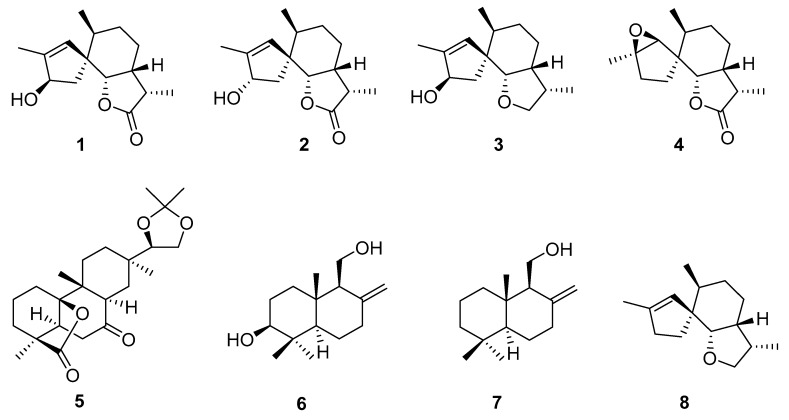
Terpenoids from *C. versicolor.*

Like all other mushrooms, the fruiting body of *C. versicolor* is harvested for its nutritional and medicinal values. The bracket or shelf mushroom body in the wild or the mycelial biomass collected from the submerged fermentation could all be used for this purpose. In addition to the major macromolecules (proteins, carbohydrates, and lipids) and minerals, the fungus is known to contain potential pharmacologically active secondary metabolites belonging to small molecular weight compounds. The study by Wang et al. [5] reported the isolation of four new spiroaxane sesquiterpenes (Figure 2), tramspiroins A–D (1–4), one new rosenonolactone 15,16-acetonide (5), and the known drimane sesquiterpenes isodrimenediol (6) and funatrol D (7) from the cultures. Readers should bear in mind that these compounds isolated from the ethyl acetate fraction are non-polar and are not expected to be available in the polysaccharide fractions of the fungus (see below). Janjušević et al. [6] studied the phenolic composition of the fruiting body of *C. versicolor* of European origin. In their HPLC–MS/MS-based study, they identified 38 phenolic compounds belonging to the flavonoid (flavones, flavonols, flavanone, flavanols, biflavonoids, isoflavonoids) and hydroxy cinnamic acids. Although the ethanol and methanol extracts are generally the richest sources of these phenolic compounds, the water extracts were also shown to contain (μg/g dry weight) considerable amount of baicalein (21.60), baicalin (10.7), quercetin (31.20), isorhamnetin (14.60), catechin (17.20), amentoflavone (17.20), *p*-hydroxybenzoic acid (141.00) and cyclohexanecarboxylic acid (80.40). The biological activities of the water extracts of *C. versicolor*, especially in the antioxidant area, must, therefore, account for the cumulative effects of the phenolic compounds. These compounds are, however, not established as the main components of the fungus, and further research is required to establish their potential contribution to the known biological activities of *C. versicolor.*

### 2.2. Polysaccharides

Like other edible mushrooms, the fruiting body of *C. versicolor* is composed of carbohydrates, proteins, amino acids, and minerals. The main bioactive components of *C. versicolor* are the polysaccharopeptides (PSPs), which are isolated from the mycelium as well as fermentation broth. As a commercial product, the main sources of these PSPs are China and Japan that produce them from the strains of “COV-1” (PSP in China) and “CM-101” (polysaccharide K (PSP Krestin or PSK, in Japan), respectively. Both products have been approved as medicines primarily as adjuvants in cancer therapy. Given that over 100 strains of the fungi are known to occur, one must recognise the diversity of these products coming from different genetic and environmental sources, including the in vitro culture conditions of their mycelial production. They are made from polysaccharides covalently bonded to peptides through *O*- or *N*-glycosidic bonds. Numerous studies have established that D-glucose is the principal monosaccharide of PSP and PSK, although other sugars such as arabinose and rhamnose are also found in small amounts (e.g., [6,7,8]). One noticeable difference in these products could be the composition of polysaccharide:peptide ratio, and their relative molecular weight. The (PSK and PSP) are both proteoglucans of about 100 kDa with variations in the individual sugar compositions such as glucose, fucose, galactose, mannose, and xylose.

The distinction between the extracellular (EPS) and intracellular polysaccharides have also been made with respect to their backbone structure [7,8]. The EPS contains small amounts of galactose (Gal), mannose (Man), arabinose (Ara), xylose (Xyl) and predominantly glucose (Glc) and are composed of β-(1→3) and β-(1→6)-linked D-glucose molecules. On the other hand, PSP and PSK contain α-(1→4) and β-(1→3) glucosidic linkages in their polysaccharide moieties. D-glucose is the major monosaccharide present while fucose (Fru), Gal, Man, and Xyl are the other principal monosaccharides in PSK. Earlier studies [9] established the distinctive features of these two polysaccharides with the presence of fucose in the PSK and rhamnose and arabinose in PSP. Analysis of the polysaccharide moiety of PSP, showed the predominance of 1→4, 1→2 and 1→3 glucose linkages (molar ratio 3:1:2), together with small amounts of 1→3, 1→4 and 1→6 Gal, 1→3 and 1→6 Man, and 1→3 and 1→4 Ara linkages [9]. On the other hand, the peptide moiety of PSP contains 18 different amino acids, with aspartic and glutamic acid residues being most predominant [8]. More importantly, PSK and PSP polymers are soluble in water.

The complexity of *C. versicolor* can be seen from the detailed structural analysis, as shown for PSP-1b1 backbone by Wang et al. [10] as follows: “→4)-α-Gal*p*-(1→4)-α-Gal*p*-(1→2)-α-Man*p*-(1→4)-α-Gal*p*-(1→2)-α-Man*p*-(1→4)-α-Gal*p*-(1→4)-α-Gal*p*-(1→2)-α-Man*p*-(1→4)-α-Gal*p*-(1→2)-α-Man*p*-(1→4)→, with branches of α-1,6-Man*p*, β-1,6-Glc*p*, β-1,3,6-Glc*p*, α-1,3-Man*p*, α-1,6-Gal*p*, α-1,3-Fuc*p*, T-α-Glc*p* and T-α-Gal*p* on the *O*-6 position of α-Man*p* of the main chain, and secondary branches linked to the *O*-6 position of β-Glc*p* (β-glucose-pyranose(*p*)) of the major branch.” Awadasseid et al. [11] also isolated a water-soluble glucan extracted from *C. versicolor* called CVG with the general backbone structure of [→6)−*α*−D−Glc*p*−(1→] n. In comparison to the PSK and PSP, CVG was small with a molecular weight of 8.8 Kda and carbohydrate composition of D-Fuc, D-Ara, D-Man, D-Gal and D-Glc, in a molar ratio of 1.0/1.1/3.0/3.9/350.7, respectively. The polysaccharide isolated by Zhang et al. [12] called β-1→3 was with the main chain consisting of β -D-1,4-Glc and β-D-1,3-Glc, and branch chains situated at β-D-1,3,6-Glc and β-D-1,4,6-Glc. More research is, however, required to identify the structures of all polysaccharides from this fungus.

## 3. Anticancer Effect through Direct Toxicity to Cancer Cells

Studies during the 1990s established that *C. versicolor* polysaccharides such as PSK could inhibit hepatic carcinogenesis in rats induced by 3′-methyl-4-dimethylaminoazobenzene [13]. The direct effect of PSK on gene expression profile in cancer cells was also established back in the 1980s [14]. Studies on combination therapy with radiation further showed the increased survival rate of mice bearing MM46 tumours [15]. Corriolan as a β-(1→3) polysaccharide with some (1→6) and no (1→4) branched glucan from *C. versicolor* was shown to be effective (100 mg/kg for 30 days) in suppressing sarcoma 180 tumours in mice [16]. Since then, the direct anticancer effect of *C. versicolor* polysaccharides has been demonstrated in the various experimental models in vitro, in vivo and clinical trials (see below).

### 3.1. Evidence of Efficacy through In Vitro Studies

The direct toxicity of *C. versicolor* polysaccharide preparations to cancer/tumour cells has been demonstrated in the various in vitro models [17,18,19,20,21,22,23,24,25,26,27,28,29,30,31,32,33,34,35,36,37,38,39,40,41,42,43,44,45,46,47,48,49] (Table 1). The number of cancer types that could be targeted by the polysaccharides is incredibly large and include breast (e.g., MCF-7, HBL-100, T-47D, ZR-75-30, MDA-MB-231 and Walker 256) [18,20,32,37,44,46], lung (e.g., A-549, and SWi573) [20,21], melanoma (e.g., SKMel-188 and B16) [17,31], colon (e.g., LoVo, HT-29, SW480, WiDr, LS174 and LS174-T) [19,20,21,22,23,24,25,26,48], leukaemia (e.g., Jukart, K562, THP-1, OCI-AML3, HL-60 and U937) [20,22,24,25,26,28,30,35,36,38,39,40,48,49], cervix (e.g., HeLa [20,21], gastric cancer (e.g., AGS, KATOIII, SCG-7901 [24,25,26,48]), prostate (e.g., PC-3, JCA-1, LNCaP, DU-145 [29,43], glioma (e.g., C6) [42], hepatoma (e.g., HepG2) [45,48], and ovarian (e.g., H4-II-E) [47] cancers. The vast majority of studies are on the two known commercially available *C. versicolor* polysaccharides, PSP and PSK (Table 1), while others include small polypeptide of about 10 Kd [48], refined polysaccharide peptide fractions [23,45], and aqueous or alcoholic extract [19,20,21,31,35,37,41,43]. Given the high molecular weight nature of the polysaccharides and even most are crude products, their observed anticancer activity mostly demonstrated in less than 1 mg/mL concentrations should be considered good but many experiments even showed activity at concentrations less or equal to 100 µg/mL [17,19,24,25,26,28,32,36,38,41,46,48]. The inhibition of cell proliferation by *C. versicolor* is associated with cell cycle arrest which could vary depending on the concentration and cell type. For example, disruption of cell cycle progression and arrest at G0 phase [22], G0/G1 phase [31,33] or G1/S and G2/M phases [36] have been reported. As a mode of cell death, induction of apoptosis has been shown for many cell types which was associated with caspase-3 activation via the mitochondrial pathway [24,25,26,28,36,39].

As expected for apoptosis-inducing agents, genes and proteins that are associated with cancer cell survival (anti-apoptotic BCL-2*,* Bcl-xL, survivin) are shown to be suppressed while those markers of apoptosis (proapoptotic Bax) induction are upregulated by the *C. versicolor* polysaccharide preparations [18,32,35,36]. Induction of the intracellular level of reactive oxygen species (ROS) in cancer cells is a well-established mechanism of cell death by chemotherapeutic agents and this appears to be the case for *C. versicolor* in several cell lines [17,46]. The critical cell growth and death regulator mitogen-activated protein kinase (MAPK) is involved in the induction of cell death by *C. versicolor* polysaccharides, as shown by the enhancement of p38 MAPK phosphorylation [24,25,26,28]. Accordingly, the cytotoxicity of these polysaccharides in melanoma cells could be abolished in the presence of c-Jun N-terminal kinase (JNK) inhibitors [17] or the p38 MAPK inhibitor [24,25,26]. Key transcription factors that are involved in cancer development and metastasis could be inhibited by *C. versicolor* polysaccharides. This includes the well-defined cancer modulator, NF-κB, or its induced protein product, cyclooxygenase-2 (COX-2) [36,40]. The potential combination of *C. versicolor* polysaccharides with conventional chemotherapeutic agents has been demonstrated in vitro, as shown for camptothecin [30], doxorubicin and etoposide [32,38], and cisplatin [46]. Even at concentrations where direct toxicity was not observed, inhibition of cell migration and invasion was evident along with inhibition of key angiogenic enzymes such as matrix metalloprotease (MMP)-9 [23] or MMP-2 [19].

### 3.2. Evidence of Efficacy through Animal Models

Many in vitro experiments that showed promising effects in direct cytotoxicity study were also extended to animal models of tumour-bearing mice. This was based on the injection of the cancer cells into mice and assess the size and spread (metastasis) of the tumour in the presence or absence of *C. versicolor* polysaccharides. Table 2 has a good summary of these data with the description of the polysaccharides, their route of administration and main outcomes [14,23,29,31,37,42,45,50,51,52,53,54,55]. It appears that *C. versicolor* polysaccharides in the form of PSP, PSK, refined fractions, water or aqueous extracts exhibit anticancer effects in vivo when administered by either oral (p.o.), intraperitoneal (i.p.) or intravenous (i.v.) routs. In a combination approach, favourable responses were obtained with metronomic zoledronic acid [50] and docetaxel–taxane [51]. In addition to a reduction in the size and volume of the implanted tumours, the incidence of tumours [29,45] and angiogenesis via vascular endothelial cell growth factor (VEGF) expression have been shown to be inhibited.

### 3.3. Evidence of Efficacy through Clinical Trials

Chay et al. [56] employed a human study on *C. versicolor* extract by recruiting fifteen eligible cases of hepatocellular carcinoma patients in Singapore who failed or were unfit for standard therapy. The randomised placebo-controlled trial using 2.4 g as a daily treatment for ~5.9 weeks) showed a better quality of life without a significant difference in primary endpoint measure of the median time to progression. On the other hand, a pilot study of randomised, double-blind, and multidose study on dogs (not humans) revealed that treatment with PSP (e.g., 100 mg/kg capsules daily) could delay the progression of metastases of canine hemangiosarcoma [57]. The systematic review and meta-analysis studies by Eliza et al. [58] assessed the survival outcome in cancer patients from 13 clinical trials on *C. versicolor*. They reported an impressive result showing a significant survival advantage when compared with standard conventional anti-cancer agents alone. For example, a 9% absolute reduction in 5-year mortality was recorded with one additional patient alive for every 11 patients treated. They also reported a better 5-year survival rate in patients receiving combination treatment in cases of breast cancer, gastric cancer, or colorectal cancer. Database on ClinicalTrial.gov shows one terminated clinical trial on the potential benefit of *C. versicolor* for hepatocellular carcinoma and one currently recruiting for a vaginal gel based on *C. versicolor* medical device (PAPILOCARE) as a phase III trial. A further entry in this database is the USA (University of Minnesota), trial on *C. versicolor extract in* Stage I, Stage II, or Stage III breast cancer who have finished radiation therapy.

## 4. Anti-Cancer Effect Via Immunostimulation

Studies on the immunotherapeutic potential of the *C. versicolor* polysaccharides in cancer started in the late 1970s and accelerated in the 1980s and 1990s. In 1977, Kataoka et al. [59] reported that immuno-resistance in mice could be induced when protein-bound polysaccharides are administered together with L1210 murine leukemic cells. The suppression of TNF-α production in mice by cytotoxic antitumour agents (5-fluorouracil, cyclophosphamide and bleomycin) was shown to be ameliorated by PSK with an implication of immunotherapy potential [60]. The immunosuppressive effect of cyclophosphamide in rats could also be abolished by PSP [61]. Myelosuppressed mice due to chemotherapy could also be reversed by PSK, particularly when used in combination with granulocyte colony-stimulating factor (G-CSF), granulocyte/macrophage colony-stimulating factor (GM-CSF) or IL-3 [62]. These general immunostimulations or ameliorations of immunosuppression under cancer and depressed immune systems, either by cancer, splenectomy or other experimental agents, have been observed for *C. versicolor* polysaccharides [63,64,65,66,67,68,69].

Further studies in vitro showed the direct lymphocyte proliferative effect of PSP, while in mice, it reversed the inhibition of IL-2 production induced by cyclophosphamide along with restoration of the T-cell-mediated response [70]. The study by Kanoh et al. [71] also demonstrated that PSK could enhance the anti-tumour effects of IgG2a monoclonal antibody in the human colon cancer cell line, colo 205, both in vitro and in vivo via antibody-dependent macrophage-mediated cytotoxicity. Studies on PSK using mice bearing syngeneic plasmacytoma X5563 also showed that it enhances anti-tumour immunity by ameliorating the immunosuppressive activity of serum from tumour-bearing mice [72,73]. The tumour-induced immunosuppression could also be abolished by PSK in various cancer models in vivo [74]. Earlier in vitro studies further confirmed the direct effect of the polysaccharides on peritoneal macrophages [75], namely, interleukin-1 production by human peripheral blood mononuclear cells [76]. Further insight into the immunotherapeutic potential of *C. versicolor* polysaccharides is outlined below under the headings of in vitro, in vivo and human studies.

### 4.1. Evidence of Immunotherapy Potential through In Vitro Studies

Perhaps the best characterised pharmacological activity of *C. versicolor* relates to its immunostimulatory effects. Some of the key outcomes from in vitro studies with implications to cancer are shown in Table 3 [8,49,77,78,79,80,81,82,83,84,85,86,87,88,89,90,91,92,93,94]. The proliferative effect of the polysaccharides on mononuclear cells, such as lymphocytes [78,79,86,92], monocytes [85] or macrophages [77] and others, including splenocytes [81], has been shown for the polysaccharides. The immunostimulatory effect also includes activation of immune cells, as shown in the LPS-induced cytokine (interleukin (IL)-1β and IL-6) expression by peripheral blood mononuclear cells (PBMCs) [77] or by blood lymphocytes) [78]. Enhancement of antibody production such as IgM and IgG1 by splenocytes was reported [82], while activation of dendritic cells was evident from the expression level of surface markers in mature cells [84]. Increased level of IgM production in B cells by PSK has also been reported. Similarly, cytokines expression, including upregulation of TNF-α expression, leads to enhanced breast cancer cell killing) [79]. The production of IL-10 in mouse B cells could be enhanced by up to 60-fold for some preparations [86], while the antibody-mediated cytotoxicity of natural killer (NK) cells against cancer cells could be enhanced through IL-12-dependent and independent mechanisms [87]. Selective induction of cytokines expression that promotes Th1 and Th2 lymphocytes have been shown [92]. Also, increased nitric oxide (NO) production in polymorphonuclear cells (PMNs) or mononuclear cells such as U937 and THP-1 have been reported for PSK and its fractions [49,94].

Considerable levels of research have been devoted to understanding how *C. versicolor* polysaccharides interact with the immune cells. One of the established recognition sites for the polysaccharides is the toll-like receptors (TLRs), of which effects via TLR4 are well-documented. In mouse peritoneal macrophages, the expression of cytokines and NF-κB activation by PSP was shown to be coupled with TLR4 activation [81]. Furthermore, the induction of TNF-α and IL-6 secretion in J774A.1 cells and primary splenocytes by PSP via TLR4 has also been well established and correlated with its effect on NF-κB p65 transcription and phosphorylation of c-Jun [88]. The expression of both TLR4 and TLR5 by PSP was shown in PBMCs of human origin, while TLR9 and TLR10 appear to be downregulated [89]. Fractionation of PSK further led to the identification of two motifs: a β-glucan recognised by the Dectin-1 receptor and lipid fraction with agonistic activity towards TLR2.

### 4.2. Evidence of Immunotherapy Potential through In Vivo Studies

The animal studies on *C. versicolor* polysaccharides also support the general immunostimulatory effect (Table 4) [23,31,48,78,81,84,87,95,96,97,98,99,100,101,102,103,104]. Increased cytokine and ROS production and NF-κB activation have been reported in rats [95]. Through IL-10-dependent mechanism, an enhancement of cytokine production that was associated with T helper (Th2 and Th17 cells) (e.g., IL-2, -4, -6, -10, -17A and IFN-α and -γ) were observed for a glucan product of *C. versicolor* in cancer-bearing mice [96]. By increasing the level of IL-6, PSP could also increase the duration of endotoxin fever in rats [98]. The proinflammatory effect of *C. versicolor* is also evident from in vivo effect of PSP in inducing a writhing response in animals, which was associated with induction of the release of prostaglandin-E2 (PGE2), TNF-α, IL-1β, and histamine from macrophages and mast cells [101]. In agreement with the in vitro experiments, combination with acacia gum resulted in a selective increase in IgG level in mice treated by PSP while the IgA or IgE levels were not affected [97]. Small peptide fractions of the polysaccharides have also shown to increase IgG level in vivo as well as white blood cell (WBC) count in tumour-bearing nude mice [48]. The animal studies in rats also suggest that high temperature exposure (hyperthermia) could suppress the cytokine production by *C. versicolor* polysaccharides [78]. Furthermore, PSP has been shown to rapidly lower temperature in rats by elevating the level of TNFα [99].

The correlation between TLR4 activation by PSP and anti-tumour potential was studied in mice. This was substantiated from the fact that its anti-tumour effect and increased thymus index and spleen index were evident in tumour-bearing C57BL/10J (TLR4^+/+^) mice but not in C57BL/10ScCr (TLR4^−^) mice [81]. PSK could also enlarge lymph nodes, activate dendritic cells, and stimulate T-cells to produce cytokines, including IFN-γ, IL-2, and TNF-α [84]. The methanol extract of *C. versicolor* also induced a higher level of tumouricidal potential of peritoneal macrophage, as revealed by the study in mice subjected to melanoma cancer [31].

The beneficial effect of PSK in combination treatment with docetaxel in prostate-carrying mice was shown to be associated with immunostimulatory effects. In this case, the number of WBCs count under the combination treatment was much more favourable than docetaxel alone [51]. The potentiation effect of PSK in anti-HER2/neu mAb therapy of Neu transgenic mice with cancer was reported [87]. The potential application of PSP in potentiating radiation therapy has also been investigated where increased lymphocyte and granulocyte counts in the blood and spleen tissues were observed [102].

### 4.3. Evidence of Immunotherapy Potential through Human Studies

Perhaps the most promising immunostimulatory effect of *C. versicolor* polysaccharides resides on the reported promise in human cancer patients. In breast cancer patients, for example, PSP has been shown to upregulate cytokine genes for L-12, IL-6 and TNF-α in PBMCs [104]. A comprehensive study with 349 gastric cancer patients receiving PSK (3 g/day) as adjuvant immunotherapy also revealed a greatly improved 3-year recurrence-free survival (RFS) rates when patients were MHC class I-negative [105]. A freeze-dried mycelial powder preparation of the fungus was also reported to show the trend of increased lymphocyte counts when applied at 6 and 9 g/day doses [106]. Although only 9 women were involved in this experiment, dose-related increases in CD8^+^ T-cells and CD19^+^ B-cells (not CD4^+^ T-cells or CD16^+^56^+^ NK cells) were reported. The application of PSK in gastric cancer —(Stage II/III) studied using large group (138 patients)—further revealed a relapse-free survival rate after post-operation or when compared to oral fluorinated pyrimidine anti-metabolites alone or in combination [107]. The double-blind placebo-controlled randomised trial study by Tsang et al. [108] employed 34 patients who had completed conventional treatment for advanced non-small cell lung cancer. They showed that PSP capsules of 340 mg each, 3× daily for 4 weeks, could lead to an improvement in blood leukocyte and neutrophil counts, serum IgG and IgM. Finally, Zhong et al. [109] undertook a meta-analysis study on randomised controlled trials of *C. versicolor* along with others. They reported that the treatment had a favorable effect on elevated levels of CD3 and CD4. Earlier studies in human cancer patients also substantiate this argument [110,111,112,113].

## 5. Other Benefits of *C. versicolor* Polysaccharides

Given oxidative stress is a prominent feature in cancer patients and experimental animals transplanted with tumours, the benefits of *C. versicolor* polysaccharides have also been tested as antioxidants. In both rats bearing with Walker 256 fibrosarcoma and human cancer patients, oral administration of PSK (daily dose of 3.0 g in humans and 50 mg/kg in rats) could normalise the disease-associated oxidative stress [114]. The immunostimulatory effect of *C. versicolor* polysaccharides has also been shown to be associated with increased superoxide dismutase (SOD) activities of lymphocytes and the thymus [115]. It is also worth noting that *C. versicolor* polysaccharides have been shown to ameliorate obesity [116] or experimental diabetes in rodents [117]. As anti-inflammatory agents, they further showed their benefit in experimental animal models of osteoarthritis [118], inflammatory bowel disease [119] or induction of analgesia [120]. Their organ protective effect was also proven through experimental models of alcoholic liver injury [10] and diabetic cardiomyopathy [121]. Their immunomodulatory effect in cancer is also extended in defenses against bacteria, including against intracellular parasites such as *Neisseria gonorrhoeae* [122].

While *C. versicolor* is regarded as an edible and medicinal mushroom, there is no report on sever toxicity induced by the fungus in humans. Experiments in rats using the standardised water extract has shown no mortality and signs of toxicity in acute and sub-chronic toxicity (up to 28 days) studies for doses up to 5000 mg/kg (p.o.) [123]. Monoclonal antibody against PSK has been developed [124], and, in principle, such antibodies could reduce the long-term use of the peptide-bound polysaccharides. For the doses indicated in the various animal experiments and human studies indicated herein, the toxicity of *C. versicolor* polysaccharides is not suggested as a concern.

## 6. General Summary and Conclusions

**Figure 3:**
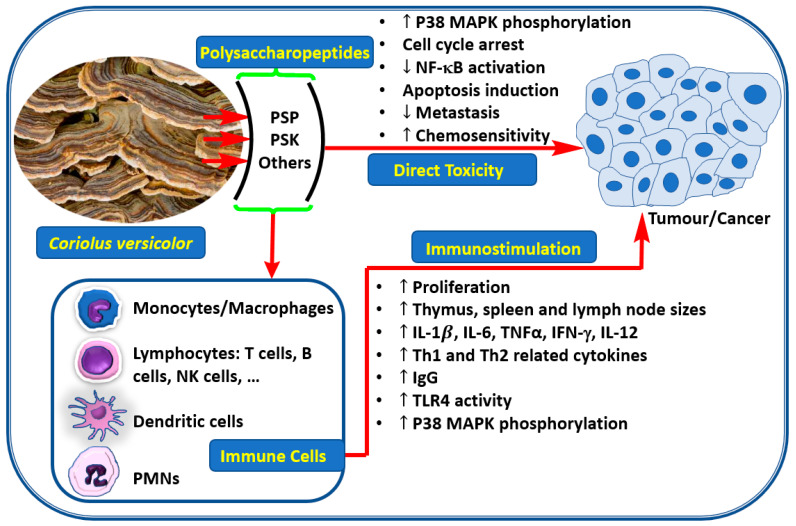
Anti-cancer potential of *C. versivolor* polysaccharides.

A great deal of attention has been given to medicinal mushrooms in recent years, with emphasis to their polysaccharide-active components. Most of these fungi are highly exploited as commercial products in far eastern countries such as Japan and China. In this regard, the edible mushroom *Dictyophora indusiata* (Vent. Ex. Pers.) Fischer (Syn. *Phallus indusiatus*) as a source of polysaccharides with main components as β-(→3)-D-glucan with side branches of β-(1→6)-glucosyl units have been established. Their chemistry, along with potential applications in cancer and immunotherapy, inflammatory and CNS diseases, among others, have been reviewed [2]. Another excellent example of the potential application of fungal polysaccharides in cancer therapy was demonstrated for *Ganoderma* *species,* which is reviewed by Cao et al. [125]. Similarly, PSP, PSK as well as other polysaccharides from *C. versicolor* have now been established to induce direct cytotoxicity to cancer/tumour cells. They also increase the release of cytokines such as TNF-α with direct implication to tumour cell killing. The overall, anti-cancer pharmacology of these polysaccharides through a direct effect on cancer cells and an indirect effect via immunostimulation is depicted in Figure 3.

Overall, the polysaccharides of *C. versicolor* have been shown to induce direct cell growth inhibitory effect and apoptosis in cancer cells. Cell cycle arrest, even in some cases at concentrations lower than 100 µg/mL in vitro, has been observed. This moderate level of activity should be considered significant since the active components are large molecular weight compounds or mixtures. Given that carbohydrates taken through the oral route are subjected to hydrolysis by intestinal enzymes, there is always a question of whether they could maintain their therapeutic value *in vivo*. Interestingly, *C. versicolor* polysaccharides, including PSP and PSK, have been shown to demonstrate anti-cancer effect in vivo following oral administration. The other well-established mechanism of the anti-cancer effect by *C. versicolor* is via immunostimulant action, as evidenced by their ability to increase the production of cytokines such as IL-12, which is Th1 related. Th-lymphocyte subsets, including Th1, Th2, Th17 or Treg, mainly through the production of key cytokines and lymphocyte subsets (B-cells, CD4^+^ and CD8^+^ T-cells, NK cells, and different stages of differentiated T-cells), have been extensively studied for their response to *C. versicolor* polysaccharides. The further induction of cytokines such as IFN-γ in T cells was evident, which, together with TNF-α, induce cancer/tumour killing. Other cytokines, including IL-1 and IL-6, have been shown to be augmented by the polysaccharides. All these events appear to enhance antibody production in T-cells while enhancing the activity of other mononuclear cells, including monocytes/macrophages.

By interacting via the membrane Ig (B-cell antigen receptor) and TLR4, *C. versicolor* polysaccharides have been shown to activate B-cells via the phosphorylation of ERK 1/2 and p38 MAPK [82]. In human PBMCs, for example, PSP activates cells through TLRs family (e.g., TLR4, TLR5, TLR6 and TLR7) and their adaptor proteins (e.g., TICAM2, HRAS, HSPA4, HSPA6, and PELI2) leading to genes activation for key cytokines (including IFN-γ, G-CSF, GM-CSF, IL-1α, IL-6) and NF-κB and TRAF6 [89]. In the latter case, it appears that PSP appears to involve the TRAM-TRIF-TRAF6 pathway of immunomodulation. With TRAM acting as a bridge between TLRs (e.g., TLR4) and TRIF6, activation of mononuclear cells to orchestrate an inflammatory response has been well-established [126]. Other important cell surface receptors for the polysaccharides include the TLR2, and Dectin-1, which are shown to be linked to the immunogenic activity of PSK [80]. Dendritic cells being an important component of the immune system, they appear to be the target for *C. versicolor* polysaccharides. For example, PSK as an adjuvant to vaccines has been demonstrated to induce the production of cytokines (e.g., IL-12, TNF-α, and IL6) in these cells both in vitro and in vivo [84]. Hence, the immunostimulatory effect coupled with direct toxicity to cancer cells by *C. versicolor* polysaccharides implies application even more than an adjuvant therapy. The evidence for signal transduction pathways, including that for TLR4 as well as other cell surface recognition markers of the polysaccharides (e.g., Dectin-1 as a β-glucan receptor), are evolving current research. The structural moieties of the polysaccharides that attribute to the various pharmacological effects also need further research.

## Figures and Tables

**Table 1:** Direct cytotoxic effects in vitro.

Preparation	Experimental Model	Key Findings	References
Protein-bound polysaccharides	Human SKMel-188 melanoma cells—100 and 200 μg/mL	Induces caspase-independent cytotoxicity; increases the intracellular level of ROS—effect inhibited by SP600125 (JNK inhibitor); cytotoxic effect abolished by receptor-interacting serine/threonine-protein kinase 1 inhibitor.	Pawlikowska et al. 2020 [17]
Immobilised fungal laccase on pH-responsive (and charge-switchable) Pluronic-stabilised silver nanoparticles (AgNPs^Trp^)	MCF-7 breast cancer cells	Inhibits cell proliferation through β-estradiol degradation and cell apoptosis; decreases in the mRNA levels of anti-apoptotic genes (BCL-2 and NF-kβ); increases the mRNA level of proapoptotic genes (p53).	Chauhan et al. 2019 [18]
Polysaccharide-rich extracts	Human colon carcinoma LoVo and HT-29 cells—proliferation; wound healing and invasion assays—10 or 100 µg/mL	Inhibits human colon cell proliferation and induces cytotoxicity; inhibits oncogenic potential, cell migration and invasion in colon cancer cells; suppresses MMP-2 enzyme activity; increases the expression of the E-cadherin.	Roca-Lema et al. 2019 [19]
Water extracts from mycelial biomass (strain It-1)—Russian origin—water and methanol extracts	Leukemia cell lines (Jukart, K562, and THP-1); solid tumors (A-549 and SWi573 (lung), HBL-100 and T-47D (breast), HeLa (cervix), and WiDr (colon)) cells—50 μg/mL	IC_50_ between 0.7–3.6 μg/mL—antiproliferative effect against lung and cervix tumors.	Shnyreva et al. 2018 [20]
Dried mycelia of Serbian origin—96% ethanol extract	Human cervix adenocarcinoma (HeLa), human colon carcinoma (LS174) and human lung adenocarcinoma (A549) cell lines	Cytotoxic activity with IC_50_ value between 60–90 μg/mL.	Knezevic et al. 2018 [21]
Polysaccharidic fraction, *Tramesan* (Patent number RM2012A000573)—extracted exopolysaccahride from *fungal* culture filtrate	Leukemic cell lines (human myeloid (OCI-AML3) and lymphoid (Jurkat) cell lines) and primary cells from AML patients—0.5–2 mg/mL	No cytotoxic effect on mononuclear cells from healthy donors; dose-dependent increase in G0 phase of cancer cells; decreases in both G1 and S phases; time- and dose-dependent induction of apoptosis in cancer cells	Ricciardi et al. 2017 [22]
Aqueous extract	Mouse mammary carcinoma 4T1 cells—0.125–2 mg/mL	No direct toxicity but inhibits cell migration and invasion; suppresses enzyme activities and protein levels of MMP-9	Luo et al. 2014 [23]
PSK	Human malignant cell lines (WiDr, HT29, SW480, KATOIII, AGS, HL-60 and U937)—30–100 μg/mL	Antiproliferative—most potent against HL-60 cells; activates caspase-3 and induces p38 MAPK phosphorylation; co-treatment with SB203580 (A p38 MAPK inhibitor) blocked apoptosis induction, caspase-3 activation and growth inhibition; apoptosis induction via mitochondrial pathway (effect on mitochondrial depolarization reversed by SB203580).	Hirahara et al. 2011, 2012, 2013 [24,25,26]
Ethanolic extracts	Human promyelocytic HL-60 cells	Suppresses cell growth; induces apoptosis; downregulates the phosphorylation of Rb; increases PARP cleavage; better effect in combination with *Ganoderma lucidum*.	Hsieh et al. 2013 [27]
PSK	HL-60 cells—100 μg/mL	Induces apoptosis without inducing cell differentiation; induces p38 MAPK phosphorylation; effect on induction of apoptosis, caspase-3 activation and growth inhibition abolished by SB203580 (p38 MAPK inhibitor).	Wang et al. 2012 [28]
PSP—Commercial source	Prostate cancer cell line PC-3—250 or 500 µg/mL	Suppresses PC-3 cell growth and in spheroid formation assay; see Table 2 for in vivo effect.	Luk et al. 2011 [29]
Polysaccharopeptide (PSP)—Commercial source—Winsor Health Products Ltd, Hong Kong	HL-60–25 μg/mL	Reduces cell proliferation; inhibits cell progression through both S and G2 phase; reduces ^3^H-thymidine uptake and prolonged DNA synthesis time; enhances the cytotoxicity of camptothecin; no effect on normal human peripheral blood mononuclear cells.	Wan et al. 2010 [30]
Methanol extract of fruiting body of Serbian origin	B16 mouse melanoma cells—200 µg/mL	Induces cell cycle arrest in the G0/G1 phase, followed by both apoptotic and secondary necrotic cell death; see Table 2 for in vivo effect.	Harhaji et al. 2008 [31]
PSP	Human breast cancer (ZR-75-30) cells—50 μg/mL or with 5 μM of doxorubicin, etoposide or cytarabine	Enhances the cytotoxicity of doxorubicin and etoposide but not cytarabine; effect associated with S-phase trap; reduces the ratio of protein expression of Bcl-xL/Bax.	Wan et al. 2008 [32]
PSK	B16, A549, Hela, AGS, Jurkat, B9 and Ando-2 tumour cell lines—50 or 100 μg/mL	Inhibits cell growth; induces cell cycle arrest, with cell accumulation in G0/G1 phase; induces apoptosis and increases caspase-3 expression.	Jimenez-Medina et al. 2008 [33]
PSP	HepG2 cells	Non-toxic dose of PSP enhanced the cytotoxicity of cyclophosphamide; decreased cell viability by 22% at 10 µg/mL	Chan and Yeung 2006 [34]
Standardised aqueous ethanol extract	HL-60 cells	Suppresses cell proliferation in a dose-dependent manner (IC_50_ = 150.6 µg/mL); increases nucleosome production from apoptotic cells; increases Bax and downregulates Bcl-2 or increases Bax/Bcl-2 proteins ratio; increases the release of cytochrome-c from mitochondria to cytosol; other effects, see Table 2	Ho et al. 2006 [35]
PSP	Human leukemia HL-60 and U-937 cells—0.1–1 mg/mL	Inhibits cell proliferation and induces apoptosis; cell type-dependent disruption of the G1/S and G2/M phases of cell cycle progression; more cytotoxic to HL-60 cells; suppresses the expression of bcl-2 and survivin while increasing Bax and cytochrome-c; enhances cleavage of PARP from its native 112-kDa form to the 89-kDa truncated product; decreases in p65 and to a lesser degree p50 forms of NF-κB; reduces the expression of COX-2.	Hsieh et al. 2006 [36]
Standardised aqueous ethanol extract—commercial source, Hong Kong	MDA-MB-231, MCF-7 and T-47D cells—400 or 600 µg/mL	Suppresses cell proliferation—IC_50_ values in ascending order of T-47D, MCF-7, MDA-MB-231, and BT-20 least affected; increases nucleosome productions in apoptotic cells; downregulates Bcl-2 protein expression (MCF-7 and T-47D cells, but not in MDA-MB-231 cells); upregulates p53 protein only in T-47D cells	Ho et al. 2005 [37]
Polysaccharide peptide (PSP)	Human promyelocytic leukemia HL-60 cells—25–100 µg/mL	Dose-dependently enhances cell apoptosis induced by doxorubicin and etoposide, but not cytarabine (Ara-C); enhances the apoptotic machinery of Doxo and VP-16 in a cell cycle-dependent manner; modulates the regulatory checkpoint cyclin E and caspase 3.	Hui et al. 2005 [38]
Polysaccharide peptide (PSP)	HL-60 cells	Induces apoptosis HL-60 cells but not of normal human T-lymphocytes; decrease in Bcl-2/Bax ratio, drop in mitochondrial transmembrane potential, cytochrome c release, and activation of caspase −3, −8 and −9	Yang et al. 2005 [39]
Proteins and peptide bound polysaccharides (PSP)	HL-60 cells—400 µg/mL	Induces apoptosis; phosphorylated regulation of early transcription factors (AP-1, EGR1, IER2 and IER5) and downregulates NF-κB pathways; increases apoptotic or anti-proliferation genes (GADD45A/B and TUSC2) and the decrease of a batch of phosphatase and kinase genes; alters carcinogenesis-related gene transcripts (SAT, DCT, Melan-A, uPA and cyclin E1).	Zeng et al. 2005 [40]
Ethanol–water extract—commercial source (Hong Konk)	Raji, NB-4, and HL-60 cells—50 to 800 µg/mL	Suppresses cell proliferation; no cytotoxic effect on normal liver cell line WRL (IC_50_ > 800 µg/mL); increases nucleosome productions in cancer but not in normal cells.	Lau et al. 2004 [41]
Polysaccharopeptide (PSP)	C6 rat glioma cells exposed to radiation (4 Gy)—1 mg/mL	Inhibits ^3^H-thymidine uptake; augments radiation-induced cancer cell damage though radiation efficacy did not increase.	Mao et al. 2001 [42]
Yunzhi (Windsor Wunxi)—a proprietary dietary supplement—ethanolic extracts (70%)	Hormone-responsive LNCaP and androgen-refractory JCA-1, PC-3, and DU-145 prostate cancer cells—0.5 mg/mL	Increases the levels STAT1 and STAT3 in JCA-1 but not LNCaP cells; reduces LNCaP cell growth, downregulates the levels of secreted but not intracellular prostate-specific antigen; no effect on level of the androgen receptor; less antiproliferative effect on PC-3 and DU-145 cells than LNCaP, and no effect on JCA-1 cells.	Hsieh and Wo 2001 [43]
PSK	MCF-7 cells—200 µg/mL	Inhibited DNA synthesis with IC_50_ value of 200 µg/mL.	Aoyagi et al. 1997 [44]
RPSP, a refined polysaccharide peptide fraction isolated by fast performance liquid chromatography (FPLC) from the crude powder of total peptide-bound polysaccharides of cultivated *Coriolus versicolor* Cov-1	Human hepatoma cell line (HepG2)	IC_50_ of 243 µg/mL for 3-day assay; no effect on normal human foetal hepatocytes.	Dong et al. 1996 [45]
PSK	NRK-49F (normal rat kidney) and H4-II-E ovarian cancer cells—100 µg/mL	Prevented cytotoxicity due to cisplatin toward NRK-49F, but enhanced the cytotoxicity on H4-II-E and human ovarian cancer cells; modulates cell-dependent effect on cisplatin-induced alteration in lipid peroxide and SOD activity.	Kobayashi et al. 1994 [46]
PSK	Walker 256 (fibrosarcoma) NRK-49F (rat normal kidney fibroblast), H4-II-E (rat hepatoma) and H4-II-E-C3 (rat hepatoma) cell lines—500 µg/mL	More pronounced antiproliferative effect in Walker 256 cells, which have more SOD activity; increased SOD activity in Walker 256 by 3.6 times and H_2_O_2_ by 2.56 times; no effect on CAT and GPx activity.	Kobayashi et al. 1994 [47]
Small polypeptide of about 10 Kd	HL-60 (leukaemia), LS174-T (colon), SMMU-7721 (hepatoma), and SCG-7901 (stomach)	Cytotoxicity against HL-60 (most sensitive cell line) with IC_50_ value of 30 µg/mL; more cytotoxic to leukemia and SCG-7901 cells than PSP or PSK.	Yang et al. 1992 [48]
PSK and four PSK subfractions	TNF-induced cytotoxicity in mouse L-929 fibroblast; interferon-γ-induced differentiation of human myelogenous leukemic U-937 and THP-1 cells.	Enhances the TNF-induced cytotoxicity against L-929 cells; induces cell differentiation; induces the expression of NBT-reducing and α-naphthyl acetate esterase activity; polysaccharides of over 200 kDa had the most potent stimulating activity.	Kim et al. 1990 [49]

Abbreviations: CAT, catalase; COX-2, cyclooxygenase 2; GPx, glutathione peroxidase; JNK, c-Jun N-terminal kinase; MAPK, mitogen-activated kinase; MMP, matrix metalloproteinase; NBT, nitroblue tetrazolium; NF-κB, nuclear factor κB; PARP, poly(ADP-ribose) polymerase; ROS, reactive oxygen species; SOD, superoxide dismutase; STAT, signal transducer and activator family of transcription.

**Table 2:** Direct antitumour effect in vivo.

Preparation	Experimental Model	Key Findings	References
Water extract of commercial source	Nude mice inoculated with human breast cancer cells - aqueous extract, metronomic zoledronic acid, or the combination of both for 4 week—1g/kg extract, p.o. daily), metronomic zoledronic acid group (0.0125 mg/kg, i.p. injected twice a week), or in combination.	Combination with metronomic zoledronic acid diminished tumor growth without increasing the incidence of lung and liver metastasis; combination therapy reserved the integrity of bones.	Ko et al. 2017 [50]
Aqueous extract	Mouse mammary carcinoma 4T1 tumour-bearing mice—1 g/kg, p.o. for 4 weeks	Decreased tumor weight by 36%, lung metastasis by 70.8%; protects bones from cancer-induced bone loss	Luo et al. 2014 [23]
PSK	Combination with taxanes for prostate transgenic adenocarcinoma of the mouse prostate (TRAMP)—C2-bearing mice—PSK with docetaxel—Mouse prostate tumor (TRAMP-C2) cells injected orthotopically—docetaxel (5 mg/kg, i.p. twice weekly); PSK (300 mg/kg daily p.o.) or in combination for 11–13 days	The combination increased more tumour suppression than either treatment alone—reduced tumor proliferation and enhanced apoptosis; other effects on immunomodulation (see Table 4).	Wenner et al. 2012 [51]
BreastDefend (BD)—extract that also contains several other mushrooms and herbal products	MDA-MB-231 cells implanted in female nude mice—100 mg/kg, ig., for 33 days.	Reduces tumour volume and anti-metastatic activity to the lungs; downregulates the expression of *PLAU* (uPA protein) and *CXCR4 genes* in breast tumors; no effect on genes associated with breast-to-lung cancer metastasis: ezrin (*EZR*), *HRAS*, *S100A4*, *CDKN1A* (protein p21) and *HTATIP2* (protein TIP30).	Jiang et al. 2012 [52]
PSP	Transgenic mice (TgMAP) mice that spontaneously develop prostate tumors—200 or 300 mg/kg p.o. 5 days per week for 20 weeks	Suppress tumourogenicity–chemopreventive property; see Table 1 for in vitro effect.	Luk et al. 2011 [29]
Methanol extract of fruiting body of Serbian origin	C57BL/6 mice inoculated with syngeneic B16 tumor cells—50 mg/kg, i.p. for 14 days	Inhibits tumor growth; peritoneal macrophages collected 21 days after tumor implantation; see Table 1 and Table 4 for other effects.	Harhaji et al. 2008 [31]
Standardised aqueous ethanol extract	Athymic nude mouse with HL-60 leukaemic xenograft model—100 mg/kg, p.o. for 28 days	Inhibits tumour growth; see Table 1 for in vitro effect.	Ho et al. 2006 [35]
VPS, a hot water extract	Swiss mice—as a 2% dose in the powdered diet for life and 1,2-dimethylhydrazine dihydrochloride (1,2-DMH) injection	No inhibitory effect on the development of large intestinal cancers; intestinal tumours and the total number of these tumors in the intestine not significantly different.	Coles et al. 2005 [53]
PSP	S180 tumor-bearing mouse model—murine sarcoma S180 cells implanted in subcutaneously in the back of each mouse—PSP solution in drinking water (35 μg/day/mouse) for 20 days	Suppress the expression of VEGF and angiogenesis and tumour markers.	Ho et al. 2004 [54]
PSP	Tumour bearing mice—radiation (8 Gy/mouse) or with PSP, i.p. 5 days before implantation and for 10 days after	Increase natural killer cell, lymphocyte and granulocyte counts in blood and spleen; no direct tumor reducing effect; see Table 1 for direct cytotoxic effect.	Mao et al. 2001 [42]
RPSP, a refined polysaccharide peptide fraction isolated by fast performance liquid chromatography from the crude powder of total peptide-bound polysaccharides of cultivated Coriolus versicolor Cov-1	Sarcoma 180 inoculated nude mice—1 mg, i.p. for 15 days	Reduces incidences of tumor growth; suppresses tumor mass; no pathological lesions in vital organs of animals such as heart, liver, spleen, lung and kidney.	Dong et al. 1996 [45]
PSK	N-methyl-N-nitrosourea-induced mammary gland tumors in rats—250 mg/kg twice a week for 3 weeks after tumour development	Inhibits tumour size and carcinogenesis	Fujii et al. 1995 [55]
PSK	Rat ascites hepatoma cell line (AH66) inoculated i.p. in rats—250 mg/kg, i.p. for 5 days before inoculation and 7 days after.	Direct effect on the transcription and translation of genens (pPIC1, pPIC2 and pPDC1).	Hirose et al. 1985 [14]

Abbreviations: VEGF, vascular endothelial cell growth factor.

**Table 3:** Immunomodulatory effects related to cancer: in vitro studies.

Preparation	Experimental Model	Key Findings	References
PSP	Normal and LPS-stimulated rat peripheral blood mononuclear cells (PBMCs—5–300 μg/mL	Enhances mitogenic activity and attenuates the induced cytokines (interleukin (IL)-1β and IL-6) production in stimulated macrophages; increases cell proliferation and pro-inflammatory cytokines release in unstimulated (LPS-free) macrophages.	Jedrzejewski et al. 2016 [77]
Protein-bound polysaccharides (PBP)	Blood lymphocytes and breast cancer cells (MCF-7)—100 and 300 μg/mL	Induces proliferative response on blood lymphocytes, as well as IL-1β and IL-6 mRNA expression; temperature of 39.5 °C blocks the PBP-induced cytotoxicity against MCF-7 cells, which correlates with reduction in TNFα level; see Table 4 for in vivo effect.	Pawlikowska et al. 2016 [78]
PSP	Breast cancer (MCF-7) cells and blood lymphocytes—100 μg/mL	Reduces cell growth; upregulates TNF-α- expression but not IL-1β and IL-6; enhances the proliferative response of blood lymphocytes associated with IL-6 and IL-1β mRNA upregulation.	Kowalczewska et al. 2016 [79]
PSK—isolation of TLR2 agonist activity from soluble β-glucan fraction—labeled the soluble β-glucan with fluorescein	Uptake of the labeled β-glucan in J774A macrophages and JAWSII dendritic cells—10–1000 µg/mL	Uptake inhibited by anti-Dectin-1 antibody but not by anti-TLR2 antibody; Dectin-1 is the receptor for β-glucan; lipid fraction enhances the uptake of the soluble β-glucan.	Quayle et al. 2015 [80]
PSP	Peritoneal macrophages from mice—25 μg/mL	Stimulates the expressions of cytokines, as well as TLR4, TRAF6, phosphorylation of NF-κB p65 and phosphorylation of c-Jun (a component of the transcription factor AP-1) in peritoneal macrophages from C57BL/10J (TLR4^+/+^) mice but not from C57BL/10ScCr (TLR4^−/−^) mice; see Table 4 for in vivo effect.	Wang et al. 2015 [81]
Polysaccharides—hot water extraction in house	Mouse splenocytes—high dose of 30 mg/mL	Stimulates splenocytes proliferation; fluorescence-labeled polysaccharides selectively stained mouse B cells but not T-cells; induces the production of IgM and IgG1 with or without exogenously added IL-4; membrane Ig (B cell antigen-receptor) acts as the polysaccharide binding protein; induces B-cell proliferation (inhibited by anti-mouse immunoglobulin (Ig) blocking antibody or in cells from TLR4-mutant mice; increases the phosphorylation of ERK-1/2 and p38 MAPK; enhances the nuclear translocation of the cytosolic NF-κB p65 subunit.	Yang et al. 2015 [82]
PSK as TLR2 agonist	PBMCs from healthy human donors—monocyte-derived DCs and tumor fusion cells	Upregulates MHC (class II and CD86) expression on DC/tumor; increases fusion efficiency; increases production of fusions derived IL-12p70; activates CD4^+^ and CD8^+^ T-cells to induce IFN-γ production; enhances induction of CTL activity specific for Mucin 1.	Koido et al. 2013 [83]
PSK	Mouse bone marrow-derived dendritic cells (DC)—5, 10, 20, 40, and 80 µg/mL)	Induces DC maturation—dose-dependent increase in the expression of CD80, CD86, MHCII, and CD40; induces the production (mRNA and protein levels) of IL-12, TNF-α, and IL-6.	Engel et al. 2013 [84]
PSP	PBMCs—10 and 100 μg/mL	Increases monocytes counts (CD14^+^/CD16^−^) compared to controls—confirmed by CD14 and MHCII antibodies; no significant effect on proliferation of T-cells, NK, and B-cells.	Sekhon et al. 2013 [85]
Purified new protein—YZP is a 12-kDa non-glycosylated protein comprising 139 amino acids, including an 18-amino acids signal peptide	Mice lymphocyte proliferation—20 μg/mL	Induced a greater than 60-fold increase in IL-10 secretion in mice B lymphocytes; specifically triggers the differentiation of CD1d^+^ B cells into IL-10-producing regulatory B cells (Bregs); enhances the expression of CD1d; activates Breg function via interaction with TLR2 and TLR4 and upregulation of the TLR-mediated signaling pathway.	Kuan et al. 2013 [86]
PSK	Human peripheral blood mononuclear cells—12–100 µg/mL	Activates NK cells to produce IFN-γ and to lyse K562 target cells; enhances trastuzumab-mediated antibody-dependent cell-mediated cytotoxicity ADCC against SKBR3 and MDA-MB-231 breast cancer cells; effect related to both direct and IL-12-dependent (indirect) mechanism.	Lu et al. 2011 [87]
PSK	J774A.1 cells and primary splenocytes—125 μg/mL	Induces TNF-α and IL-6 secretion by wild-type but not by TLR4-deficient peritoneal macrophages; TNFα secretion by J774A.1 cells and primary splenocytes effect inhibited by TLR4 blocking antibody.	Price et al. 2010 [88]
PSP	Human PBMCs—25 μg/mL	Upregulates the expression of (e.g., IFN-γ, CXCL10, TLR4, TLR5) while downregulating (e.g., TLR9, TLR10, SARM1, TOLLIP) other genes related with TLR signaling pathway; upregulated some cytokines (GCSF, GM-CSF, IL-1α, IL-6, IFN-γ) by more than 1.3 times; increases the mRNA levels of TRAM, TRIF, and TRAF6; increases the protein level of TRAF6.	Li et al. 2010 [89]
PSK	B-cells—human B-cell line BALL-1—1–100 µg/mL	Enhances IgM production in B-cells.	Maruyama et al. 2009 [90]
Polysaccharides from New Zealand isolate (Wr-74) and a patented strain (ATCC-20545) of *C. versicolor—*culture medium isolates	Murine splenocytes—extracellular polysaccharide (1150 µg/mL), and intracellular polysaccharide (IPS) (100 µg/mL)	Induces cytokine production (interleukin 12 and gamma interferon) in murine splenocytes.	Cui et al. 2007 [8]
PSP	Human T lymphocyte proliferation—100 or 500 µg/mL	Exhibits similar and additive inhibitory effects to ciclosporin to suppress activated T-cell proliferation, Th1 cytokines; reduces CD3^+^/CD25^+^ cell expression but not Th2 cytokine expression.	Lee et al. 2008 [91]
Ethanol–water extract—commercial source	Proliferation of murine (BALB/c mice) splenic lymphocytes—12.5–400 μg/mL	Enhances cell proliferation by up to 2.4-fold in a time- and dose-dependent manner; upregulates Th1-related cytokines (IL-2 and IL-12); enhanced the level of Th1-related cytokines (IFN-γ and IL-18) transiently (24 h, but not at 48 and 72 h) while Th2-(IL-4 and IL-6).	Ho et al. 2004 [92]
PSK	Dendritic cells derived from CD14-positive cells obtained from human peripheral blood monocytes	Increases the expression of HLA (class II antigen) and CD40; increases the number and expression of CD80-, CD86- and CD83-positive cells; decreases FITC-dextran uptake; augments IL-12 production and allogeneic mixed lymphocyte reaction; induces antigen-specific cytotoxicity.	Kanazawa et al. 2004 [93]
PSK	Mouse peritoneal PMNs—500 µg/ml	In combination with IFN-γ, increases NO production.	Asai et al. 2000 [94]
PSK and fractions (F1 <50 kDa; F2 50–100 kDa; F3 100–200 kDa; F4 >200 kDa)	U937 and THP-1 cells differentiation; TNF-induced cytotoxicity in L929 cells—5–500 µg/mL	In combination with IFN-γ, increases NO production and cell differentiation; enhances cytotoxicity in L929 cells; fraction F4 is the most active.	Kim et al. 1990 [49]

Abbreviations: ADCC, antibody-dependent cellular cytotoxicity; CTL, cytotoxic T lymphocytes; DC, dendritic cells; FITC, fluorescein isothiocyanate; HLA, human leukocyte antigen; IFN-γ, interferon-γ; LPS, lipopolysaccharides; MHC, major histocompatibility complex; PMBCs, peripheral blood mononuclear cells; PMN, polymorphonuclear cells; SARM, sterile-alpha and Armadillo motif-containing protein; TOLLIP, Toll interacting protein; TRIF, TIR domain-containing adaptor protein-inducing interferon β; TRAM, (TRIF)–related adaptor molecule; TRAF, tumor necrosis factor receptor (TNF-R)-associated factor.

**Table 4:** Immunomodulatory effects related to cancer: in vivo studies.

Preparation	Experimental Model	Key Findings	References
Extract from *Coriolus versicolor* (Cov 1 strain)	Pre-injection in LPS-treated rats and PBMCs isolated—100 mg/kg, i.p.	Partially prevents endotoxin tolerance through maintaining febrile response; increases IL-6 and greater NF-κB activation in response to LPS stimulation ex vivo; enhances mitogenic effect of LPS and increases ROS generation.	Jedrzejewski et al. 2019 [95]
Glucan—home-made purification—[→6)-α-D- Glcp-(1→]_n_.	Sarcoma 180-bearing mice—100 or 200 mg/kg for nine days, subcutaneously	Promotes the secretion of IL-2, −4, −6, −10, −17A and IFN-α and -γ; enhances cytokine production associated with T-helper Th2 and Th17 cells; effect dependent on IL-10.	Awadasseid et al. 2017 [96]
PSP	C57BL/6 male mice—50 mg/kg, p.o.	When combined with acacia gum, increased total IgG titre levels (day 4) while decreasing IgM titre had no effect on IgA or IgE titre levels.	Sekhon et al. 2016 [97]
Protein-bound polysaccharides (PBP)	Fever-range hyperthermia (FRH) combined with PBP in rats—100 mg/kg i.p.	Combination treatment of (FRH + PBP) decrease IL-1β, IL-6 and TNF-α mRNA expression in peripheral blood mononuclear cells; see Table 3 for in vitro effect.	Pawlikowska et al. 2016 [78]
PSP	Male Wistar rats—100 mg/kg, i.p. 2 h before LPS	Increases the duration of endotoxin fever; increases the blood level of IL-6 (3 or 14 h post-injection); effect inhibited by anti-IL-6 antibody (30 µg/rat).	Jedrzejewski et al. 2015 [98]
PSP	500 mg/kg/d by p.o. in mice for 25 days	Decreases the mean weights of tumors; increases thymus index and spleen index relative in tumour-bearing C57BL/10J (TLR4^+/+^) mice but not in C57BL/10ScCr (TLR4^−^) mice; see Table 3 for in vitro effect.	Wang et al. 2015 [81]
PSP	Male Wistar rats—50, 100 and 200 mg/kg, i.p.	Induces a rapid reduction in temperature; elevates TNF-α level; anti-TNF-α antibody abolish effect on temperature.	Jedrzejewski et al. 2014 [99]
Aqueous extract	Mouse mammary carcinoma 4T1 tumor bearing mice—1 g/kg, p.o. for 4 weeks	Increases IL-2, 6, 12, TNF-α and IFN-γ productions from the spleen lymphocytes; see Table 1 and Table 2 for other effects	Luo et al. 2014 [23]
PSK	As an adjuvant to OVAp323-339 vaccine in vivo—DC activation 1000 µg—one injection by intradermal route	Enlarges draining lymph nodes with higher number of activated DC; stimulates the proliferation of OVA-specific T-cells, and induces T-cells that produce multiple cytokines (IFN-γ, IL-2, and TNF-α; see Table 3 for in vitro effect.	Engel et al. 2013 [84]
PSK	PSK with docetaxel- mouse prostate tumor (TRAMP-C2) cells injected orthotopically—docetaxel (5 mg/kg) injected i.p. twice weekly; PSK (300 mg/kg) daily by oral gavage or combination for 11–13 days	Lower level of decrease in number of white blood cells than docetaxel alone; increases numbers of tumor-infiltrating CD4+ and CD8+ T-cells; PSK with or without docetaxel enhance mRNA expression of IFN-γ—no effect on T-regulatory FoxP3 mRNA expression in tumors; augments the docetaxel-induced splenic natural killer cell cytolytic activity against YAC-1 target cells.	Wenner et al. 2012 [51]
PSK	Neu transgenic mice received subcutaneous implant of 1 million MMC cells—100 mg/kg, p.o. 3 times per week for up to 4 weeks	Potentiates the anti-tumour effect of anti-HER2/neu mAb therapy in neu-T mice; see Table 3 for in vitro effect.	Lu et al. 2011 [87]
Methanol extract of fruiting body of Serbian origin	C57BL/6 mice inoculated with syngeneic B16 tumor cells—50 mg/kg, i.p. for 14 days	Peritoneal macrophages collected 21 days after tumor implantation possess stronger tumouristatic activity ex vivo than those from untreated animals; see Table 1 and Table 2 for other effects.	Harhaji et al. 2008 [31]
PSP—composed of 90% polysaccharides (74.6% glucose, 2.7% galactose, 1.5% mannose, 2.4% fucose and 4.8% xylose) and 10% peptides (18 different amino acids, mostly aspartic acid and glutamic acid)	Acetic acid-induced writhing model—0.2–2 μmol/kg, i.p. in hot-plate test; 2–4 μmol/kg, i.p. in acetic acid-induced writhing response; 0.05–4 μmol/kg, i.p. induction of writhing response by itself.	Decreased the number of acetic acid-induced writhing by 92.9%; PSP itself induces a dose-dependent writhing response; increased the release of PGE2, TNF-α, IL-1β, and histamine in mouse peritoneal macrophages and mast cells both in vivo and in vitro (1–100 μM).	Chan et al. 2006 [100]
Purified polysaccharide (CV-S2-Fr.I) of *C. versicolor* obtained by Sepharose CL-6B gel chromatography	Mouse peritoneal macrophage—100 µg/mL	Enhanced macrophage lysosomal enzyme activity by 250%; enhances the induction of NO production by interferon-γ (no effect by its own).	Jeong et al. 2006 [101]
PSP	Tumour bearing mice—radiation (8 Gy/mouse) or with PSP, i.p. 5 days before implantation and for 10 days after	Increases natural killer cell, lymphocyte and granulocyte counts in blood and spleen; no direct tumor reducing effect; see Table 1 for direct cytotoxic effect.	Mao et al. 2001 [102]
PSP	C57BL/6NIA mice—diets containing 0.1, 0.5 or 1.0% PSP for 1 month	No effect on mitogenic response to Con A, PHA or LPS, or on production of IL-1, IL-2, IL- 4 and PGE2; induced higher delayed-type hypersensitivity response (1.0% PSP) in old but not in young mice.	Wu et al. 1998 [103]
Small polypeptide of about 10 Kd	Human tumour cells (SMMU-7721 or LS174-T) inculated into nude mice—2 mg, i.p. for 2 weeks.	Increases WBC and IgG levels; decreases the incidence of tumor mass.	Yang et al. 1992 [48]

Abbreviations: Con A, concanavalin A; IFN-γ, interferon-γ; LPS, lipopolysaccharide; NO, nitric oxide; PGE2, prostaglandin E2; PHA, phytohemagglutinin; WBC, white blood cell.
